# Viscosity measurement of Xanthan–Poly(vinyl alcohol) mixture and its effect on the mechanical properties of the hydrogel for 3D modeling

**DOI:** 10.1038/s41598-018-34986-4

**Published:** 2018-11-08

**Authors:** Yasutomo Shimizu, Tadao Tanabe, Hiroshi Yoshida, Motohiro Kasuya, Tadao Matsunaga, Yoichi Haga, Kazue Kurihara, Makoto Ohta

**Affiliations:** 1 Institute of Fluid Science, Tohoku University 2-1-1, Katahira, Aoba-ku, Sendai, Miyagi 980-8577 Japan; 2 Graduate School of Engineering, Tohoku University 6-6 Aramaki-aza-aoba, Aoba-ku, Sendai, Miyagi 980-8579 Japan; 3 Institute of Multidisciplinary Research for Advanced Materials, Tohoku University 2-1-1, Katahira, Aoba-ku, Sendai, Miyagi 980-8577 Japan; 4 Graduate School of Biomedical Engineering, Tohoku University 6-6 Aramaki-aza-aoba, Aoba-ku, Sendai, Miyagi 980-8579 Japan

## Abstract

Biomodels made of poly(vinyl alcohol) (PVA) are demanded because they can represent the geometries and mechanical properties of human tissues realistically. Injecting and molding, commonly used in three-dimensional (3D) modeling, help to represent the blood vessels accurately. However, these techniques sometimes require higher pressures than the upper pressure limit of the dispensers for pouring in high viscosity materials; the material viscosity should therefore be lower. Moreover, the mechanical properties of the biomodels should be reproduced. This study proposes a PVA solution through the addition of xanthan gum (XG) for 3D modeling, which lowers liquid viscosity while maintaining the mechanical properties of biomodels. XG is known to facilitate the achievement of non-Newtonian fluidity; however, the effects of XG on a PVA solution and PVA hydrogel (PVA-H) are not confirmed. The viscosity measurement using 15 wt% PVA with XG solution (PVA/XG) shows that it will provide easier pouring than 17 wt% PVA solution. The tensile test using the PVA-H of PVA(15 wt%)/XG(0.2 wt%) reveals that the gel is comparable in Young’s modulus to 17 wt% PVA-H. X-ray diffraction shows the crystalline structures of the PVA/XG gel and PVA-H are identical. Thus, this PVA/XG would be useful for fabricating biomodels using injection molding techniques.

## Introduction

Poly(vinyl alcohol) (PVA) is a known material that can mimic the shape and mechanical characteristics of human soft tissues. Although living soft tissues have complex geometries and come in various sizes, hydrogels made of PVA solution (PVA-H) can easily represent both the realistic mechanical properties and geometrical structures^1^. Moreover, PVA-H models can accommodate a wide range of mechanical properties of living tissues, and this wide range can be used to represent the realistic living tissues more accurately than the range in silicone models, when we control the concentration of the PVA solution, the degree of saponification, and the molecular weight of the PVA^2^. Therefore, PVA-H models can be usually represented in the form of geometries and mechanical properties of blood vessels and these models can be used for training new medical doctors and simulate surgeries such as intravascular treatment.

Painting, dip-coating, and injection molding are common methods to fabricate biomodels^3^. Painting and dip-coating are usually necessary to control the wall thickness of the model. However, although such control is important in fabrication, it is difficult to do so accurately. Nowadays, three-dimensional (3D) modeling technology is progressing rapidly, and its benefits should be considered. In general, injection molding requires high-pressure liquid dispensers to build complex geometries and to control the wall thickness accurately based on design data. The liquid dispensers require an appropriate choice of viscosity, and this may affect the fabrication capability. Hence, the inappropriate choice could lead to an inaccurate geometrical structure because of excessive pouring and clogging in the dispensers^4^. In addition, the range of PVA-H stiffness in vessel models should be constant even if the viscosity of the PVA solution is decreased because the PVA-H mechanical properties play a very important role in biomodels. However, a method to control the PVA viscosity while maintaining constant PVA-H mechanical properties is yet to be established. Moreover, the low-viscosity PVA solution may help prevent clogging and allow the solution to be poured stably.

The purpose of this study is to establish a new PVA solution for 3D modeling. We mix xanthan gum (XG) is into the PVA solution to control the shear viscosity of the latter. XG solution is a known non-Newtonian pseudoplastic fluid^5–8^ and it has a potential to decrease its viscosity in accordance with the increase in the shear rate. In contrast, the mechanical characteristics of PVA/XG solution remain unclear. Therefore, we performed the following experiments to determine the behavior of the PVA/XG solutions and gels made from them. First, we compare the shearing viscosities in 15 wt% PVA solutions made with various XG concentrations with that in 17 wt% PVA solution that is usually prepared for vessel biomodels. This step is important for determining the influence of XG on the viscosity of PVA solution. Second, we use a tensile test to measure the Young’s modulus of each PVA-H specimen. Finally, we use X-ray diffraction (XRD) to reveal the crystal structure of each PVA-H specimen to discuss the relationship between solution viscosity and Young’s modulus.

## Results

### Shear viscosity measurement

**Figure 1:**
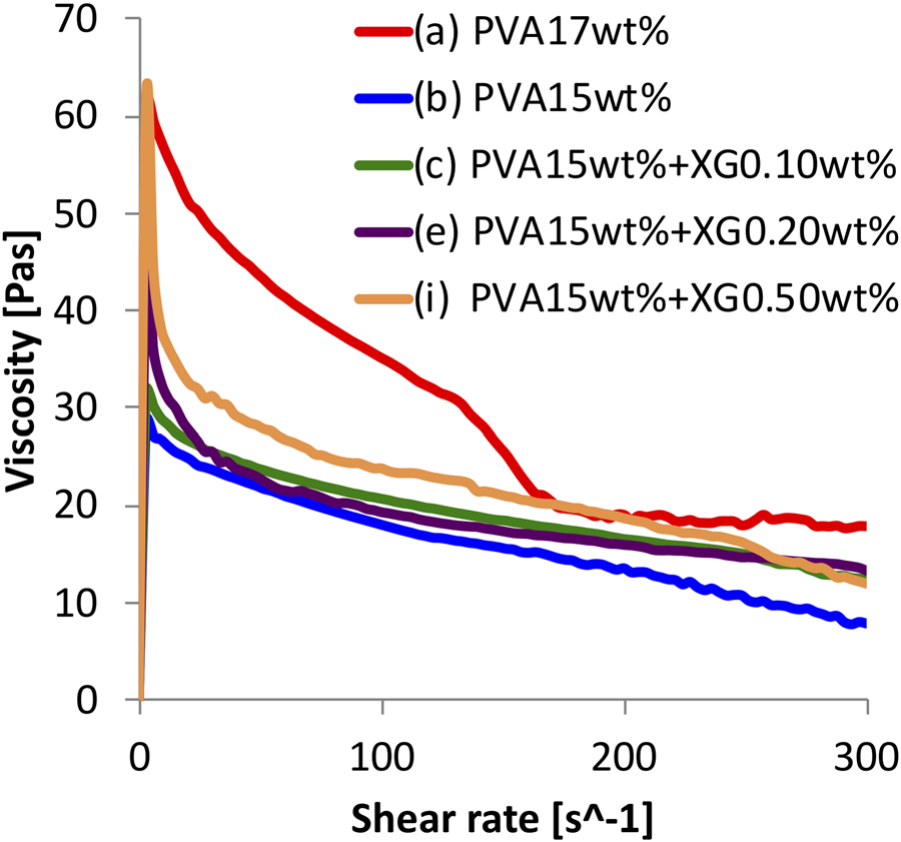
Relationship between shear viscosity and shear rate in PVA + XG solutions.

Figure 1 shows the relationship between the shear viscosity and shear rate of PVA solutions. Each solution behaved as a pseudoplastic, and this behavior has a stronger dependency on the concentration of XG. In this study, the “nominal” cut-off viscosity is defined as less than 0.1 Pa·s difference from the immediately preceding measurement point. However, the viscosity in high shear rate still decreases slightly, and this viscosity can be a threshold of the maximum pressure limit for pouring. This viscosity and the shear rate at the cut-off viscosity of each solution are shown in Table 1. The shear viscosity of each solution was the highest at the lowest shear rate, and the viscosity of the mixtures increased with XG concentration. In particular, the viscosity of solution (i) was 61.8 Pa·s which is close to the 62.1 Pa·s solution (a). The dynamic viscosities of the mixtures decreased rapidly at a low shear rate (<50 s^−1^) and decreased linearly in the region of 50–300 s^−1^. In contrast, the viscosity of the PVA solution without XG decreased less rapidly below 50^−1^ compared with those of the mixtures. The viscosity of solution (a) at 50 s^−1^ and 300 s^−1^ was 27.9 Pa·s and 6.0 Pa·s higher, respectively, than that of solution (i). These results indicate that XG works as a viscous agent in the PVA solution and that including XG greatly affects the viscosity at a low shear rate, especially below 50 s^−1^.

**Table 1:** Cut-off viscosity and the shear rate at the viscosity in Fig. 1.

Specimen code	Cut-off viscosity [Pa·s]	Shear rate [s^−1^]
(a)	19.78	175.8
(b)	16.76	121.2
(c)	19.76	118.2
(e)	19.02	109.1
(i)	24.51	84.84

The cut-off viscosity can be a threshold of the maximum pressure limit for pouring.

### Tensile test

**Figure 2:**
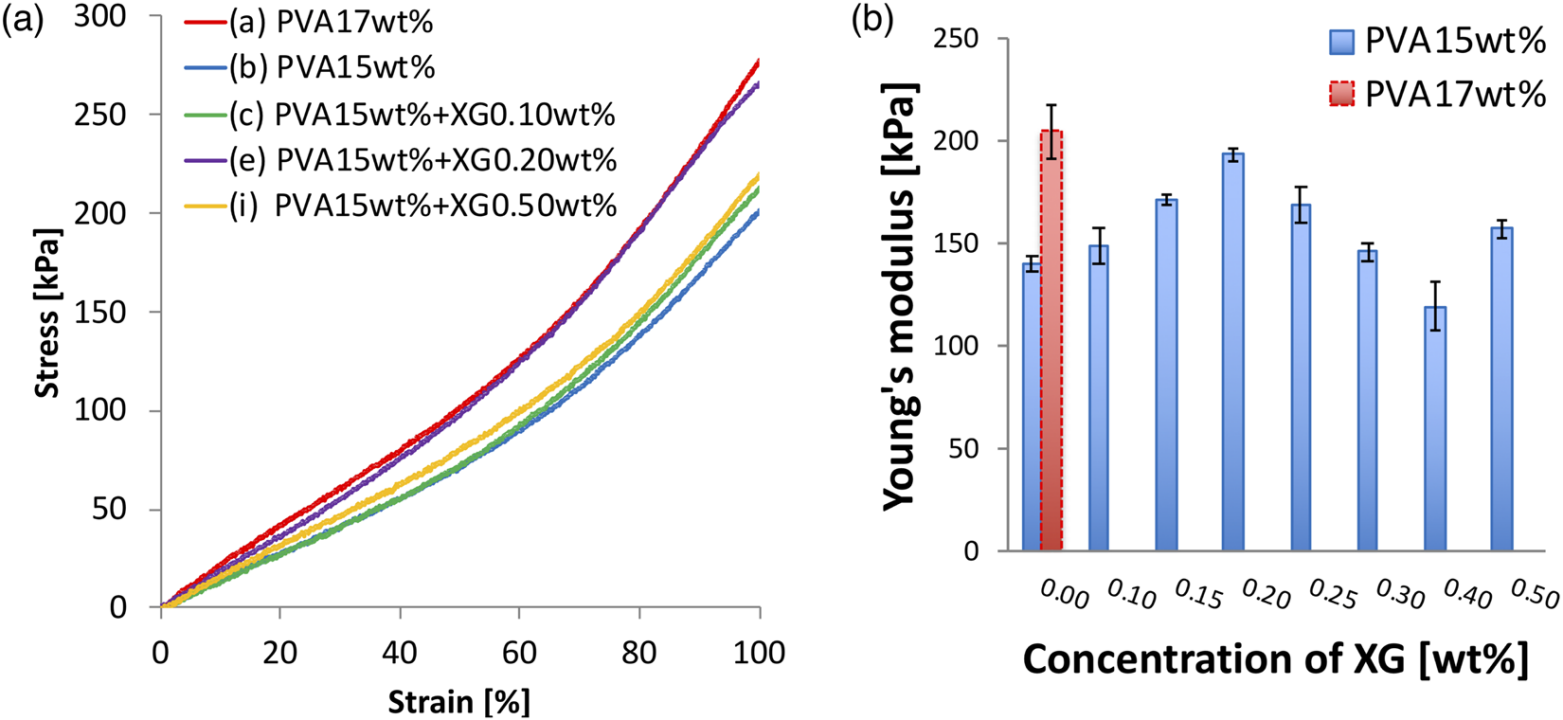
The results of the tensile test. (**a**) Stress–strain curves in tensile tests of PVA-H specimens. The Young’s modulus in each specimen is defined at 50% strain. These results show the third time measurements. (**b**) Relationship between Young’s modulus in 17 and 15 wt% PVA-H specimens and XG concentration. (n = 3, mean ± SD).

Figure 2 show the results of the tensile test. (a) shows stress–strain curve of the tensile test for each specimen and (b) shows the relationship between Young’s modulus of PVA-H and the XG concentration in the 15 wt% PVA solution. The Young’s modulus is calculated from the slope of 0–0.5 strain in the stress–strain curve. As a reference, the result for 17 wt% PVA-H is shown in Fig. 2(b) as a dotted line. The value of Young’s modulus for specimen (a) is 204.8 kPa, and the closest PVA-H value is 194.0 kPa for specimen (e).

### XRD measurement

**Figure 3:**
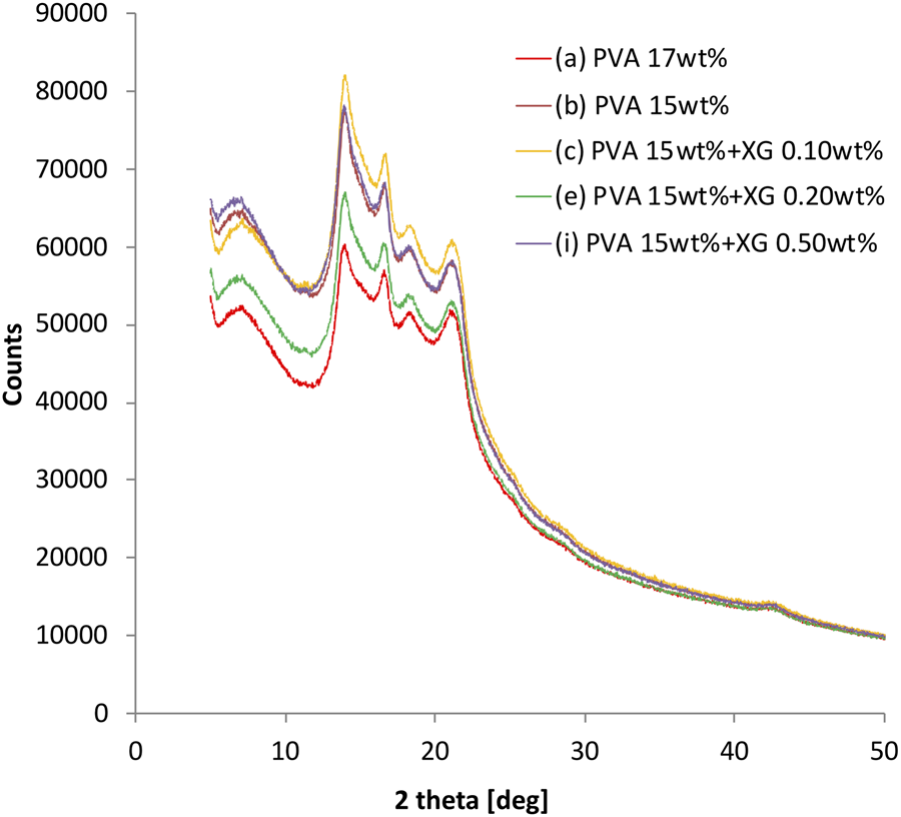
XRD profiles of PVA-H specimens wrapped by the PP film.

Figure 3 shows the XRD profiles for the PVA-H specimens wrapped by a PP film. The five 2θ peaks in each specimen were observed at approximately 7.12°, 13.96°, 16.60°, 18.24°, and 21.10° in each specimen seems to be from the PP sheet.

Regarding the profile of pure XG, a previous study has reported that it has three peaks at 16.42°, 19.64°, and 20.10°^9^. However, the peak change in the specimens was not observed around the peaks of pure XG. It means that the contained XG was not crystallized and the PVA specimens could maintain the original crystal form. These results lead that all profiles in each specimen does not dramatically change even if XG is containing.

### Fabrication of tube model

**Figure 4:**
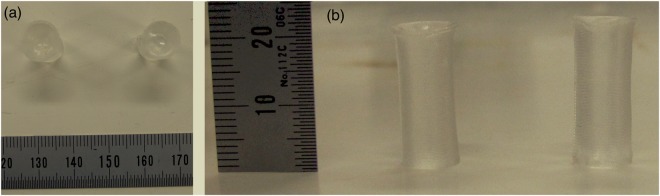
Photos of PVA-H specimens after gelation. (**a**) shows the top view and (**b**) shows side view. Left side in both figures shows the specimen (**e**) and right side in both figures shows the specimen (**a**).

Figure 4 shows the image of the fabricated tube models, especially using specimen (a) and (e) for comparison. The geometry and the transparency are completely represented and the visualization of both models are little different. The size of the specimen (a) is *ϕ*8.06 × 20.57 mm and that of the specimen (e) is *ϕ*8.02 × 20.11 mm. From the model fabrication, both specimens (a) and (e) are represented with a high accuracy and the error of the representation for both molds was less than 3%. The concave meniscus caused by the surface tension at the mold occurred in both specimens. The dent sizes are 0.90 mm of the specimen (a) and 1.06 mm of the specimen (e). The PVA solution with XG can reproduce the geometrical characteristics.

The time required for pouring the solutions to the mold was 27.0 and 16.8 s for the specimen (a) and (e). The time required for pouring the solution for specimen (e) is 10.2 s less than that to specimen (a).

## Discussion

In this study, we established a novel PVA solution to manufacture biomodels. The solution has a low shear viscosity at low shear rates (below 150 s^−1^) while being poured from a dispenser, but it maintains a high Young’s modulus of the resulting PVA-H. However, there is a trade-off between the viscosity of the solution and the Young’s modulus of the gel. XG solution is generally used as a viscous agent and can increase the static viscosity of solvent mixtures^5^. In contrast, The PVA concentration can also strongly affect both viscosity of the PVA solution and the Young’s modulus of the specimen. Thus, adding XG (especially, 0.20 wt% into 15 wt% PVA solution) and controlling PVA concentration are quite important for maintaining the original Young’s modulus of the specimen produced by the 17 wt% PVA solution.

Aqueous XG solution behaves as pseudoplastic fluid, and the shear viscosity of the solutions with XG can decrease more rapidly with a shear rate compared with that of solvents without XG. These known phenomena were observed in the present study. Therefore, we reason that this decrease in shear viscosity could help expand the range of applicability of the PVA solution and minimize damage to the dispensers.

Although the change in shear viscosity differs between the PVA solution and the mixture solution, the values of Young’s modulus of PVA-H specimens made from either solution could get close, as shown in Fig. 2(b). From the results of the tensile tests, the 17 wt% PVA-H was represented by PVA-H using the mixture solution of PVA and XG (specimens (b) through (i)), and the lowest difference in Young’s modulus was 5.3% between specimens (a) and (d). XG is known to be able to form intermolecular associations, implying that XG can increase the compressive strength of PVA-H^10^. From another perspective, XG can provide large intermolecular spaces between the polymer chains because of its structure implying that PVA-H with XG can contain much more water than it can without XG. However, this water content can decrease the Young’s modulus because of weak hydrogen bonding^11^. This unstableness of the Young’s modulus according to the XG concentration is due to the balance between the strengths of hydrogen bonding and intermolecular association in PVA-H.

XRD measurement can reveal the crystal structure of the PVA-H specimens. From our previous study, the profiles indicate the first 2θ peak around 7.12° comes from PVA-H and the others come from the PP sheet^12^. The XRD profiles of all the specimens were not dramatically changed after containing XG, indicating that the change in crystal structure is slightly affected by both XG inclusion and PVA concentration. It is known that XG in PVA can affect the viscosity of the specimens based on phase mixing and interaction which prevented the phase separation^13^. However, in this research, it is difficult to specify the relation between the viscosity change in the solutions and Young’s modulus of the specimens in this research. In other words, the other characteristics except the crystal structure might reveal this relation. For example, in future, the amount of the crystal can be measured by X-ray small angle scattering. In addition, the content of XG was ultralow volume in this research. The increase in the content might be able to show the tendency of the crystal structures.

From the model fabrication, the geometry of the mold is represented using specimens (a) and (e) and the error of representation of the mold was less than 3%. The meniscus was observed in both models and this phenomenon does not come from the material. From these results, PVA with XG solution can also have the potential of representing the geometry of molds. In addition, the viscosity decrease may enlarge the upper limit of the narrowness of the nozzle and should benefit printing with shorter time. In our results, 10.2 s were saved for a pouring from the comparison of the time using specimens (a) and (e). This time reduction is caused by the decreased in viscosity based on the effect of XG during pouring.

By adding XG powder to the low-concentration PVA solution, the resulting XG-PVA solution would be useful for 3D modeling, especially for 3D inkjet printing. This technique may be useful for making models of blood vessels and also other industrial products.

## Materials and Methods

### PVA solution and PVA-H

We dissolved PVA (JF- 17, DP = 1700, SV = 99 mol%; Japan Vam & Poval Co. Ltd., Japan) in an aqueous solution of distilled water and dimethyl sulfoxide (DMSO) (20/80 w/w; Toray Fine Chemicals Co., Ltd., Japan). DMSO is a well-known solvent for PVA solutions to prevent crystallization of the contained water and to maintain high transparency of the model, and a PVA solution containing DMSO itself also behaves as a pseudoplastic fluid^14^. We also dissolved XG (Wako Pure Chemical Industries, Ltd., Japan) in the PVA solution for the XG-mixture specimens. The solution was stirred for 2 h at 100 °C before it was cooled down to 40 °C in an oven. Subsequently, the PVA solution destined for PVA-H was poured into a mold and stored at −30 °C for 24 h to promote gelation. A dish with dimensions *ϕ*35 × 7 mm was prepared as the mold for the XRD measurements, whereas a mold with dimensions 8 × 1 × 50 mm was prepared for the tensile tests. The ratios of the mixtures prepared in this study are summarized in Table 2.

**Table 2:** Mixture ratios of PVA into DMSO aqueous solution and XG into the PVA solution.

Specimen code	PVA [wt%]	XG [wt%]
(a)	17	0
(b)	15	0
(c)	15	0.10
(d)	15	0.15
(e)	15	0.20
(f)	15	0.25
(g)	15	0.30
(h)	15	0.40
(i)	15	0.50

### Shear viscosity measurement

To observe the influence of XG on PVA viscosity, we measured the dynamic viscosities of the PVA solution and PVA/XG solution using a rheometer (RS-CPS, sensor number R25-2/3; Brookfield, Canada). The sensor had a measuring cone with a radius of 12.5 mm and an angle of 2°. For each of solutions (a–c), (e), and (i), we poured 0.3 ml onto the plate of the rheometer. Each measurement was performed at 40 °C with a shear rate of 0–300 s^−1^ in every 3 sec.

### Tensile test

We evaluated the PVA-H elasticity by measuring Young’s modulus using a uniaxial tensile tester (EZ-S; Shimadzu Co., Ltd., Japan). The specimens were prepared using solutions (a)–(i). The conditions of the tensile test were based on those in previous studies^15,16^. The distance between the upper and lower sides of the clamps to set up the specimens was set to 40 mm and each specimen was stretched at a constant speed of 20 mm/min to a strain of 1.0 and then returned to a strain of 0.0; this cycle was repeated three times. We calculated Young’s modulus of each specimen based on Hooke’s law as given by equation (1), where *σ*, *E*, and *ε* represent the shear stress, the Young’s modulus, and the strain, respectively; the Young’s modulus is determined at the slope of the strain from 0 to 0.5 in the linear range. Young’s modulus can be calculated from the slope in the elastic region. A strain of 1.0 in the plastic region is sufficient for this experiment, although there are no cracks in the specimens; this was done on the third cycle to exclude the hysteresis in PVA-H:

1 $$\sigma =E\varepsilon .$$

### XRD measurement

We performed θ–2θ measurements using an X-ray diffractometer (SmartLab; Rigaku Co. Ltd., Japan) to identify the molecular structure of the PVA-H specimens and to discuss the change in the viscosity of the PVA solution. PVA-H specimens were prepared using solutions (a)–(c), (e), and (i), and all specimens were wrapped in a polypropylene (PP) sheet to prevent evaporation of DMSO and water. Each measurement was performed at a voltage of 45 kV, a current of 200 mA, and a wavelength of 0.154 nm. We varied the 2θ angle from 5° to 50° in steps of 0.01°.

### Fabrication of tube model to evaluate the new PVA solution

A geometrical comparison was performed using specimens (a) and (e) to evaluate the influence of the solutions on the model geometries. A simple cylinder, with a diameter of 8.00 mm and a height of 20.00 mm, was fabricated for this comparison. The specimens (a) and (e) were poured using a dispenser (MS-1D, Musashi Engineering Inc., Japan) into the mold made from water soluble PVA (MELFIL, The Nippon Synthetic Chemical Industry Co. Ltd., Japan). This pouring was performed under a pressure of 0.2 MPa and using a stainless nozzle of 18 G (inner diameter of 0.84 mm, outer diameter of 1.26 mm, and a length of 13.00 mm). The specimens were frozen at −30 °C for 24 h to promote gelation after pouring. After 24 h, the PVA solution gelated and the tube model was completed. To evaluate the model quality, the sizes of the model and meniscus in the model was measured. In addition, pouring time into the mold is also measured to consider the benefit of the new solution to the fabrication.

## Conclusions

In this study, we have developed a novel method to make PVA solutions for 3D modeling. The new solution including XG (0.20 wt% XG + 15 wt% PVA) can represent a realistic Young’s modulus of blood vessels with a lower dynamic viscosity compared with the previously used PVA solution (17 wt% PVA). This fluid characteristic can be achieved using a low concentration of the PVA solution and adding XG. XG gives the PVA solution pseudoplastic characteristics and controls the Young’s modulus of the specimens, although the crystal structure of the resulting PVA-H is little affected by the addition of XG. Finally, in comparison with the previous PVA solution, the new solution maintains the accuracy of the fabricating geometry and spends shorter time for the fabrication.
